# Development and Internal Validation of a Predictive Model of Perceived Stress Among Military Students: A LASSO Regression Analysis

**DOI:** 10.3390/ijerph23060741

**Published:** 2026-06-01

**Authors:** Tamadhir Al-Mahrouqi, Mohammed Al Alawi, Alya Al Harrasi, Mohammed Al Zadjali, Atheer Al Jahwari, Siham Al Shamli, Amira Al Housni

**Affiliations:** 1 Department of Behavioral Medicine, College of Medicine and Health Sciences, Sultan Qaboos University, Muscat 123, Oman; t.almahrouqi@squ.edu.om (T.A.-M.); m.alalawi2@squ.edu.om (M.A.A.); 2 Department of Behavioral Medicine, Sultan Qaboos University Hospital, Muscat 123, Oman; alya.harrasi@gmail.com (A.A.H.);; 3 Department of Psychiatry, Medical City of Military and Security Services, Muscat 112, Oman; r20121@resident.omsb.org; 4 Psychiatry Residency Training Program, Oman Medical Specialty Board, Muscat 132, Oman; atheerjahwari99@gmail.com

**Keywords:** perceived stress, military, predictive modeling, personality traits, depression, psychological well-being

## Abstract

**Highlights:**

**Public health relevance—How does this work relate to a public health issue?**

- Perceived stress among first-year military students is a relevant public health concern because elevated stress during academic and military transition may affect psychological well-being, functioning, and adjustment.
- The study addresses early identification of students at higher risk of stress by examining psychological factors such as depressive symptoms, well-being, and personality traits.

**Public health significance—Why is this work of significance to public health?**

- The findings support the importance of preventive mental health strategies in military training environments, particularly during the early transition period.
- By identifying correlates of perceived stress, such as neuroticism, depressive symptoms, and low psychological well-being, the study may help institutions develop targeted screening and support programs.

**Public health implications—What are the key implications or messages for practitioners, policy makers and/or researchers in public health?**

- Policy makers and institutions may consider multidimensional screening at induction, including assessment of stress, depressive symptoms, psychological well-being, and relevant personality traits, followed by targeted support where needed.
- Practitioners should prioritize culturally sensitive counseling, stress-management interventions, resilience training, well-being education, and peer-support initiatives.

**Abstract:**

This study aimed to develop and internally validate a predictive model of perceived stress among first-year military male students to examine the predictive contribution of personality traits, depressive symptoms, and psychological well-being. Understanding these psychological predictors may support interventions for students at elevated risk of stress during military and academic transition. A cross-sectional web-based survey included 274 first-year male students at the Military Technological College in Oman. Outcome measures included the Perceived Stress Scale (PSS-10), the Patient Health Questionnaire (PHQ-9) for depressive symptoms, the WHO-5 Well-being Index, and the Big Five Inventory assessing personality traits. All variables were analyzed as continuous measures. Predictive modeling was performed using Least Absolute Shrinkage and Selection Operator (LASSO) linear regression with repeated 70/30 train–test splitting across 100 iterations and 10-fold cross-validation for internal validation. The final analytic sample included 266 participants after exclusion of incomplete responses. Across the 100 internal validation runs, the LASSO model accounted for approximately 40% of the variance in perceived stress (training R^2^ = 0.44 ± 0.04; test R^2^ = 0.40 ± 0.08). Neuroticism (β = 0.35) and depressive symptoms (β = 0.15) showed positive associations with perceived stress, whereas psychological well-being showed a negative association (β = −0.32). PHQ-9, WHO-5, and neuroticism were selected in 100% of the repeated LASSO models, which showed the most stable predictive contribution. Model performance on the test datasets showed stable predictive accuracy (MSE = 20.24 ± 2.48; RMSE = 4.49 ± 0.28; MAE = 3.61 ± 0.23). These findings demonstrate that personality traits, depressive symptoms, and psychological well-being collectively contribute to the statistical modeling of perceived stress among military students. The internally validated associative model may support institutional interventions for students vulnerable to elevated stress, informing targeted preventive mental health strategies within military training environments.

## 1. Introduction

Addressing stress is crucial as it affects one’s quality of life, involving physical and mental health, academic performance, and interpersonal relationships. First-year students frequently experience stress due to academic and social transitions. To better understand these challenges, stress can be categorized into three levels: personal, interpersonal, and situational [1]. Personal-level stressors include age, gender, and financial load [2]. While interpersonal-level stressors involve homesickness, loss of a supportive social circle, and having controversial relationships with their peers and the faculty [3,4]. Situational-level stressors related to one’s specialty and institution [5]. Furthermore, stress is a universal challenge for college students, military students have unique experiences with stress as they encounter additional stressors, including physical training, strict academic standards, and adaptation to military life [6]. In Oman’s collectivist culture, strong family and tribal bonds shape individuals’ responses to adversity, and psychological difficulties are often managed within the family or community network rather than through formal mental-health services. Therefore, cultural expectations and the stigma surrounding mental health may further intensify stress, as students navigate both traditional social norms and the demands of military training, such as navigating rigorous physical training, a strict chain of command, and a dual identity as both soldiers and scholars, the pressure to conform to military discipline, perform academically, and uphold unit cohesion can magnify everyday stressors.

However, not all students experience stress in the same way. Individual differences, particularly personality traits, play a crucial role in determining stress vulnerability [7]. According to the most widely accepted personality model, the big five dimensions of personality are represented through five distinct dimensions: an individual’s level of extraversion, neuroticism, agreeableness, conscientiousness, and openness to new experiences [8]. Neuroticism is a personality trait that embraces an increased predisposition to experience negative affective states, specifically anger, anxiety, irritability, and depression [9]. It was found that high trait neuroticism contributes to the development and/or the progression of various psychiatric disorders [10]. Previous studies have shown that people with higher trait neuroticism, in comparison to their counterparts, have higher baseline stress perception and heightened reports of perceived threat, and they are more likely to judge unclear situations as threatening or unfavorable [11,12]. Not to mention, higher trait neuroticism is greatly linked to the use of maladaptive coping strategies as they are having trouble dealing with stressors [13,14]. Referring to the Grounded in the Transactional Model of Stress and Coping [15], this study posits that the way individuals appraise and respond to stressors, mediated by personality traits and emotional states, plays a critical role in their overall stress levels. This theoretical perspective underlines the importance of early detection and intervention in high-stress environments such as military academies. Despite the reliable bond between neuroticism and perceptions of stress, it must be remembered that personality traits are displayed over a continuum and do not act in isolation [11].

Beyond personality, stress is closely linked to mental health outcomes, particularly depression and psychological well-being, creating a complex cycle of emotional strain. There is a complicated relationship between perceived stress and depression, where each one affects the other mutually and reciprocally in a bidirectional way. Stress can interact with depression in a variety of ways: as a comorbid condition, a consequence, a symptom, or a cause [16]. Building on this idea, the Hypothalamic–pituitary–adrenal (HPA) axis hyperactivity may imply an important pathophysiological mechanism behind depression and act as a mediator in its relationship with stress [17]. Based on the cognitive resource theory [18], processing depressed emotions may exhaust or redirect the required resources to perform control functions, including the cognitive resources, leaving them with distorted or deficient cognition to deal with surroundings, resulting in more perceived stress [19,20]. Furthermore, overthinking and rumination induced by negative emotions like depression may blunt the analytic thinking and executive function necessary to handle stressful life events, leading to profound stress levels due to inefficient processing and increased self-criticism that keep the individual from further recognizing the stress of unresolved life events [21,22]. These findings highlight the importance of developing predictive models that can identify stress early, particularly in high-risk populations such as military students [23].

The World Health Organization (WHO) defines health as a state of well-being, including physical, social, and psychological well-being [23]. Psychological well-being (PWB) can be described as the absence of mental health issues and the presence of self-acceptance, personal growth, purposeful and meaningful life, positive relationships with others, environmental mastery, and self-determination [24,25]. Researchers at Concordia University’s psychology department found that optimism, a component of PWB, can mitigate perceived stress levels [26]. Optimistic individuals tend to exhibit lower levels of cortisol, a “stress hormone,” in response to stressful situations [26]. Strong social support fosters a person’s PWB, which reduces stress levels, as was established in a cross-sectional study conducted in the Philippines during the COVID-19 pandemic [27].

There is a lack of studies examining the combined associations of personality traits, depressive symptoms, and psychological well-being with perceived stress among Omani military students. First-year military students represent a distinct group because they face simultaneous academic, physical, disciplinary, and social transitions when entering a structured military training environment. These demands may shape how stress is perceived and reported, making this population important to study separately from general university students.

Personality traits, depressive symptoms, and psychological well-being were selected because they are theoretically relevant to stress appraisal and coping. Neuroticism may be associated with greater sensitivity to stressors, depressive symptoms may co-occur with higher perceived stress, and psychological well-being may be linked to more adaptive coping resources. However, in the context of military training, these relationships may be influenced by additional demands, including strict hierarchy, regimented routines, performance expectations, and adjustment to cadet life.

Therefore, this study aimed to develop and internally validate a multivariable LASSO regression model of perceived stress among first-year military students. Stress was selected as the outcome variable because it reflects psychological strain during academic and military transition. Given the cross-sectional design, the study does not infer causal or temporal relationships; rather, it evaluates the relative contribution of personality traits, depressive symptoms, and psychological well-being to variability in perceived stress. We hypothesized that neuroticism and depressive symptoms would be positively associated with perceived stress, whereas psychological well-being and more adaptive personality traits would show negative associations. Model development incorporated cross-validation and repeated train–test splitting to improve model stability, reduce overfitting, and assess internal performance.

## 2. Methods

### 2.1. Study Design and Study Setting

This was a cross-sectional web-based research study design. The study population included all male first-year students who were enrolled in the Military Technological College (MTC) in Oman. The MTC program combines engineering education with vocational training and military activities. The college serves as a key source of skilled and trained technical personnel for the Ministry of Defense, the Sultan’s Armed Forces of Oman, and various military and security agencies across the country. In addition, MTC is the only military program in Oman that secures jobs for its graduates. This study focuses exclusively on male students because MTC enrolls only men in its military training program.

### 2.2. Data Collection

All undergraduate students in the 2024 cohort were invited to participate in the study through a gathering in the university’s main lecture hall on 1st of September 2024. The research team explained the study’s objectives, with clear indications that participation was voluntary and anonymous. A barcode linking to the online survey was displayed, allowing students to scan and complete it on their mobile phones.

### 2.3. Exclusion Criteria

The study excluded any student who was not in their first year of college. Students who did not sign the consent or provided incomplete answers were excluded.

### 2.4. Outcome Measures

The questionnaire comprised two main sections and included a total of 80 items. The first section collected demographic and background information, while the second section assessed participants’ responses using Likert-scale items. The questionnaire was administered entirely in English and developed using Google Forms. As English is the language of instruction at the MTC, students are expected to be proficient in English and able to complete the questionnaire.

### 2.5. Stress

Stress was measured using the Perceived Stress Scale (PSS), which contains 10 items to assess the extent to which individuals perceive situations in their lives as stressful [28]. The responses were rated on a 5 point Likert scale, ranging from 0 (never) to 4 (very often). High values indicate high levels of perceived stress. A systematic review of psychometric properties reported Cronbach’s alpha values for the PSS-10 ranging from 0.78 to 0.91 [29]. However, lower reliability scores have been reported in several Middle Eastern samples [30,31]. For example, a study of Emirati university students reported a Cronbach’s alpha of 0.67 [31]. In this study, the PSS-10 had a Cronbach’s alpha of 0.69.

### 2.6. Depression

Depressive symptoms are measured using the Patient Health Questionnaire-9 (PHQ-9), a widely used screening tool for assessing the severity of depression [32]. The participant will have to complete the 9-item set, which is in line with the DSM-5 diagnostic criteria for major depressive disorder. The answers are rated on a 4-point Likert scale: 0 (not at all), 1 (several days), 2 (more than half the days), 3 (nearly every day). The scale has 27 scoring possibilities, from 0 to 27, a higher value on PHQ-9 indicates greater depressive symptom severity. A study by Al-Ghafri et al. examined the PHQ-9′s effectiveness in an Omani sample and found that a cut-off score of 12 achieved the best trade-off between sensitivity (80.6%) and specificity (94%) [33]. Based on this, a cut-off score of 12 was used to signal the presence of significant depression. Moreover, the internal consistency in the current study was Cronbach Alpha 0.83, indicating good reliability.

### 2.7. Well-Being

Well-being is measured using the WHO-5 Well-being Index, which consists of five positively worded items assessing subjective psychological well-being [34]. Each item is rated on a 6-point Likert scale ranging from 0 (at no time) to 5 (all of the time). After adding all items on the scale, a total score is generated (score range: 0–25). High scores suggest great well-being. A study involving Arab patients reported that a WHO-5 cut-off value of 9.5 was the most effective threshold, ensuring 80% sensitivity and 70% specificity in depression screening [35]. The WHO-5 was found to be reliable and valid both as a screening tool for depression and as an outcome measure in clinical trials according to the literature [36]. The Cronbach’s alpha reliability in this study was 0.87. A 9.5 was used as a cut-off score.

### 2.8. Personality Traits

Personality traits are measured using the Big Five Inventory (BFI), which consists of 44 items that assess five key dimensions: openness, conscientiousness, extraversion, agreeableness, and neuroticism [37]. Each trait is evaluated through a series of statements rated on a Likert scale, typically ranging from 1 (strongly disagree) to 5 (strongly agree). The reliability coefficients for the five traits were: extraversion (α = 0.40), agreeableness (α = 0.70), conscientiousness (α = 0.68), neuroticism (α = 0.70), and openness (α = 0.59). To address the low Cronbach’s alpha values for extraversion and openness, item-total correlations were examined. In the extraversion subscale, the removal of Item 1 (Is talkative) and Item 21 (Tends to be quiet) increased the α value to 0.57. For the openness subscale, removing Item 35 (Prefers work that is routine) and Item 41 (Has few artistic interests) raised the α value to 0.75.

### 2.9. Ethical Approval

Informed consent was obtained from all participants. The ethical approval of this study was obtained from the Military Technological College Research Committee on the 12 May 2024. The approval number is WD/KAT/1008-2024. This study was conducted in accordance with the Declaration of Helsinki [38] for ethical human research.

### 2.10. Sample Size Calculation

For predictive regression, adequacy is primarily driven by the number of candidate predictors and the need to limit overfitting, with common methodological guidance recommending approximately 10–20 observations per predictor parameter and incorporating internal validation to quantify model optimism. Given the planned inclusion of a limited set of theoretically grounded psychological predictors (personality domains, depressive symptoms, and well-being), the available cohort size at the Military Technological College (approximately 300 first-year students) was expected to provide sufficient information to support stable model estimation. The achieved analytic sample (n = 266) exceeded conventional participant-to-predictor requirements and was therefore considered adequate for prediction modeling. In addition, the use of penalized regression (LASSO) with 10-fold cross-validation, alongside repeated train–test splitting (70/30) across 100 iterations, provided shrinkage and internal validation, further reducing the risk of overfitting and supporting the robustness and generalizability of the final model.

### 2.11. Data Analysis

Descriptive statistics, including frequency, percentage, mean, standard deviation (SD), median, and range, were used to explore the profile of the students according to their demographic and psychological measures. All psychometric variables (PSS, PHQ-9, WHO-5, and BFI personality domains) were analyzed as continuous measures to preserve statistical information and optimize predictive modeling performance. Pearson correlations were conducted as preliminary exploratory analyses to examine relationships between candidate predictors and perceived stress prior to model development. We used a variance inflation factor (VIF) to confirm that multicollinearity did not occur between the explanatory variables. In addition, skewness and kurtosis tests confirmed normality assumptions. The Least Absolute Shrinkage and Selection Operator (LASSO) linear regression was applied using the glmnet packages in R.

Predictors were internally standardized during model estimation according to the default settings of the glmnet package. Reported coefficients are presented on the original measurement scales following back-transformation. The full dataset was randomly split into training (70%) and testing (30%) subsets across 100 independent iterations with different random seeds. Ten-fold cross-validation was applied to the training data to identify the optimal regularization parameter (λ), which was then used to estimate the final LASSO regression model on the training data before evaluating its performance on the test data. LASSO linear regression is particularly useful because of the large number of predictors we examined as it applies a penalization that reduces coefficients of less important predictors to zero. Model performance metrics such as R^2^, mean squared error (MSE), root mean squared error (RMSE), and mean absolute error (MAE) were averaged across 100 iterations to account for variability due to random splitting. Additionally, the stability of the LASSO coefficients was assessed across the 100 repeated iterations. For each predictor, the mean coefficient, standard deviation of the coefficient, and selection frequency were calculated. Selection frequency represented the proportion of repeated models in which each predictor had a non-zero coefficient. This was done to assess whether predictors were consistently retained across random train-test splits. The predictors selected in 100% of the repeated LASSO models were then examined in a sensitivity analysis. The aim of the sensitivity analysis was to assess whether predictive performance was maintained after removal of less stable predictors. All analyses were performed in JASP and R (version 4.5.1). A value of *p*  <  0.05 was considered to be statistically significant.

## 3. Results

The study population consisted of 300 male first-year students from MTC in Oman, with a high response rate of 91% (n = 274). The participants had a mean age of 19.04 ± 1.62 years and an average high school grade of 83.55 ± 9.20. The socio-demographics of the participants are presented in Table 1. Eight participants were removed due to missing responses to specific questions. A final sample of 266 observations was used for the regression analysis.

**Figure 1:**
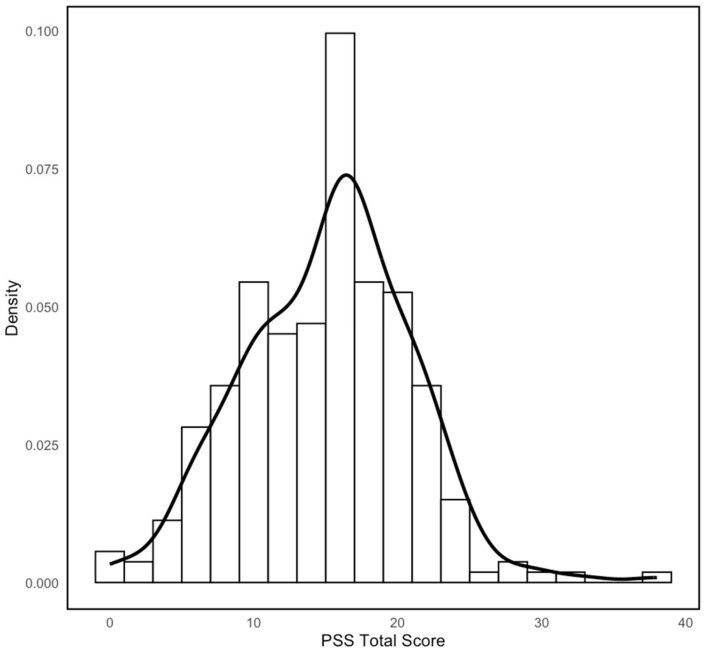
Distribution of PSS scores.

**Figure 2:**
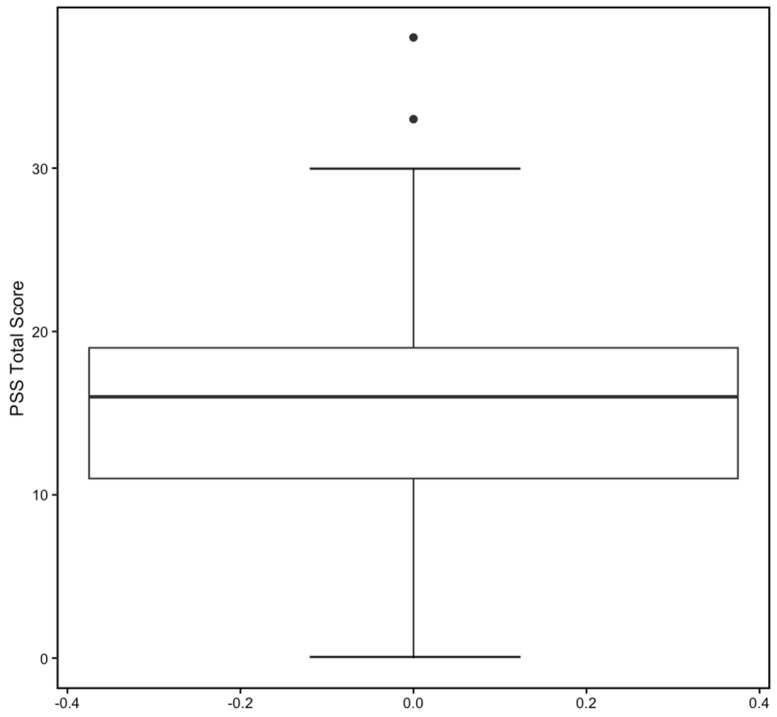
Boxplot of PSS scores.

The mean scores of the participants were: PSS (15.18 ± 5.80), WHO (15.71 ± 5.45), PHQ (8.02 ± 5.46), neuroticism (20.21 ± 5.38), conscientiousness (31.22 ± 5.74), extraversion (20.18 ± 5.38), agreeableness (33.61 ± 5.77), and openness (26.57 ± 5.78). Preliminary analyses examined data distribution and multicollinearity. The data showed normal distribution with skewness (−0.54 to 0.86) and kurtosis (−0.57 to 0.59) within acceptable ranges ±2. Tests for multicollinearity showed acceptable tolerance (0.43–0.77) and VIF values (1.30–2.35), indicating no concerning collinearity between predictors. As shown in Figure 1, the PSS-10 scores followed an approximately normal distribution with slight right skewness. The corresponding boxplot in Figure 2 shows a symmetric spread around the median and potential outliers. Detailed descriptive statistics are presented in Table 2.

Table 3 displays the results of Pearson’s correlation coefficient, which indicated a significantly positive correlation between the perceived stress and both PHQ (*r* = 0.45, *p* < 0.001) and neuroticism (*r* = 0.56, *p* < 0.001). Significant negative correlations were found with WHO (*r* = −0.53, *p* < 0.001), extraversion (*r* = −0.29, *p* < 0.001), conscientiousness (*r* = −0.38, *p* < 0.001), and agreeableness (*r* = −0.23, *p* < 0.001). No significant correlation between PSS and openness (*r* = −0.08, *p* = 0.18).

**Figure 3:**
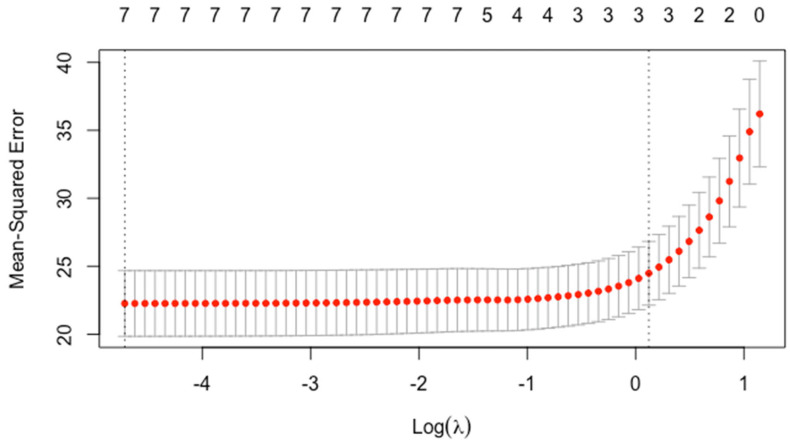
LASSO regression cross-validation curve.

**Figure 4:**
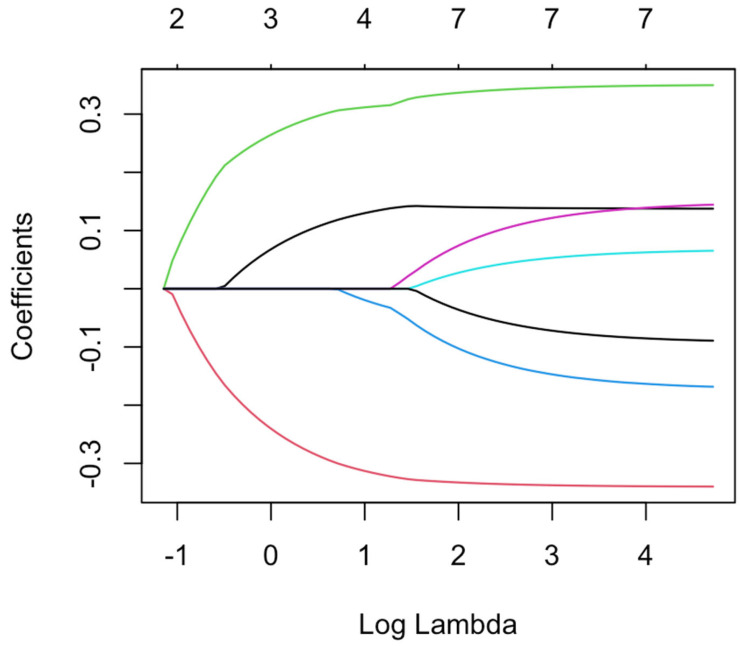
Path diagram of the LASSO coefficients.

A LASSO regression model was trained on 70% of the sample (n = 187), using 10-fold cross-validation to identify the optimal regularization parameter (λ), as illustrated in Figure 3. The remaining 30% (n = 79) served as a holdout test set to evaluate model performance. The coefficients path plot in Figure 4 is based on a single representative training set (seed = 21).

Across the 100 runs, the model explained 44% of the stress variability in the training data (R^2^ = 0.44 ± 0.04) and 40% in the test data (R^2^ = 0.40 ± 0.08). This indicates that around 40% of the variance in perceived stress was explained by personality traits, depression, and well-being. On the test data, the model showed good accuracy with an MSE of 20.24 ± 2.48, an RMSE of 4.49 ± 0.28, and an MAE of 3.61 ± 0.23.

The stability of the LASSO coefficients was examined across the 100 runs (Table 4). PHQ, WHO, and neuroticism were selected in all 100 models, so they had the most stable contribution to perceived stress. Neuroticism had the strongest positive coefficient (β = 0.35), followed by PHQ (β = 0.15). WHO had a strong negative coefficient (β = −0.32), which suggests that higher psychological well-being was associated with lower perceived stress. The other personality traits were less stable. Extraversion, openness, agreeableness, and conscientiousness were selected in fewer than half of the repeated models.

A sensitivity analysis was then conducted using only the predictors that were selected in 100% of the repeated LASSO models. These predictors were PHQ, WHO, and neuroticism. The reduced model explained 41% of the variance in perceived stress in the test data (R^2^ = 0.41 ± 0.08). The model also showed good accuracy, with an MSE of 19.85 ± 2.50, an RMSE of 4.45 ± 0.28, and an MAE of 3.57 ± 0.23 (Table 5). This suggests that most of the model’s predictive ability came from PHQ, WHO, and neuroticism.

## 4. Discussion

This study aimed to develop a model to examine the extent to which personality traits, depressive symptoms, and psychological well-being were associated with perceived stress among first-year students at the MTC in Oman. The findings showed that neuroticism and depressive symptoms were consistently associated with higher perceived stress, whereas psychological well-being was negatively associated with perceived stress. Although extraversion, agreeableness, and conscientiousness showed significant negative correlations with perceived stress, their contribution in the LASSO model was less stable. Openness was not significantly correlated with perceived stress and showed limited predictive contribution. The full LASSO model explained approximately 44% of the variance in perceived stress in the training data and 40% in the test data, indicating moderate predictive performance and reasonable generalizability. Importantly, PHQ, WHO, and neuroticism were selected in all repeated LASSO models, and the reduced sensitivity model using only these predictors showed comparable performance. These findings suggest that depressive symptoms, lower psychological well-being, and neuroticism may be useful indicators for identifying students at greater risk of perceived stress and for informing targeted stress-management strategies within military training programs.

Although most predictor directions in the LASSO model were broadly consistent with the bivariate correlations, agreeableness showed a notable exception. In the Pearson correlation analysis, agreeableness was negatively associated with perceived stress; however, its mean coefficient in the multivariable LASSO model was positive. This apparent reversal should be interpreted cautiously, as agreeableness was selected in fewer than half of the repeated models, suggesting that its independent contribution was unstable. The discrepancy may reflect shared variance with other psychological or personality variables rather than a direct positive association with perceived stress. Moreover, our findings align with previous studies that examined the association between personality traits and perceived stress. Individuals with high neuroticism traits are more sensitive to stressful situations, and they are more emotionally unstable [10]. The strong association between neuroticism and stress may be rooted in the trait’s characteristic tendency toward emotional instability and heightened sensitivity to negative stimuli. This suggests that interventions aimed at improving emotional regulation, such as cognitive–behavioral strategies, could be particularly effective for individuals scoring high on neuroticism, from the lens of the Transactional Model of Stress and Coping, high-neuroticism individuals are predisposed to appraise everyday challenges as threats rather than manageable demands, which amplifies their perceived stress and calls for CBT-based reappraisal training [10], neuroticism may be associated with stress perception through maladaptive appraisals, while well-being may be linked to adaptive coping. This demonstrates the need for interventions targeting cognitive restructuring and emotional regulation to address stress in military settings.

Consistent with prior research across diverse populations, our results reinforce the link between neuroticism and perceived stress. A study in India found similar correlations between neuroticism and perceived academic stress among college students, with no significant correlations for other personality traits [7]. Similarly, a cross-sectional study done among medical postgraduates found a positive correlation between neuroticism and perceived stress. However, unlike the present study, there was a negative association between perceived stress and conscientiousness and agreeableness [39]. Another study was carried out among Bangladeshi university students, established that four of the personality traits, agreeableness, extraversion, neuroticism, and openness together, accounted for 23.5% of the variance in perceived stress (R^2^ = 0.24), neuroticism being the most influential predictor (β = 0.40), but there was no association between conscientiousness personality trait and perceived stress [40]. However, while some studies have found additional associations (e.g., a negative link between conscientiousness and stress), our model did not reveal such relationships. This divergence may be attributed to cultural differences related to the population’s knowledge of mental health and the unique stressors inherent in military training, as mentioned before, physical training, strict academic standards, and the military lifestyle.

It is also noteworthy that certain traits, such as openness, did not demonstrate significant associations with perceived stress in this study. One explanation may be that in the highly structured and disciplined environment of military training, traits like openness, which emphasize creativity and novelty-seeking, are less relevant to stress appraisal compared to traits such as neuroticism. Similarly, while extraversion showed only a modest relationship, opportunities for social interaction and expression of extraversion may be limited by the regimented nature of cadet life, which could attenuate its protective role against stress. Cultural factors may further contribute, as personality traits developed in Western candidates may not fully capture how these constructs are expressed in Omani or broader Arab populations.

Moreover, our findings support the cognitive–emotional model of stress [16], personality traits are associated with how individuals appraise and respond to stressors. This theoretical perspective highlights the need for personalized stress management strategies in environments that demand high resilience, such as military training. Although perceived stress and depression exhibit a bidirectional relationship, there is a lack of studies examine the association between depressive symptoms and perceived stress. Cognitive Resource Theory predicts this reciprocal relationship: depressive symptoms consume executive resources needed for adaptive coping, thereby raising stress levels, which in turn can deepen depressive rumination, a longitudinal survey that included Chinese college students displayed those negative emotions (i.e., depression and anxiety) and perceived stress were associated with each other in a dynamic reciprocal pattern [16]. Regarding the association between psychological well-being and perceived stress, the results of our study are in agreement with previous research conducted among Turkish university students during the COVID-19 pandemic which found that there is a significant correlation between psychological well-being and perceived stress, where psychological well-being accounted for 0.9% of the variation in perceived stress (R^2^ = 0.009) [41]. It is possible to say that people with strong psychological well-being may perceive lower stress levels.

To our knowledge, this is the first study to examine personality traits, depression, and psychological well-being together as predictors of stress among Omani military students at MTC, and it suggests a clear, stepwise approach for training programs: first, incorporate brief neuroticism assessments at induction to flag cadets at highest risk; next, implement routine depression screening (e.g., PHQ-9) during orientation to detect early symptoms; then, offer those who screen positive targeted interventions mindfulness-based stress reduction workshops, resilience training, and culturally sensitive counseling; and finally, embed ongoing well-being initiatives into the curriculum that build emotional regulation, bolster mindfulness and resilience, and strengthen peer support networks. By following this sequence identification, screening, intervention, and sustained well-being education, military colleges can translate our predictive model into practical measures that prevent stress and enhance cadet mental health.

### Strengths and Limitations

A key strength of this study is the use of validated measures, including the PSS, PHQ, WHO, and BFI. The train–test split also provided an internal assessment of model performance.

Several limitations should be noted. First, the cross-sectional design means that the findings should be interpreted as concurrent predictive associations, not causal or directional relationships. Second, the use of self-reported data may introduce response bias. Third, the study was conducted at a single institution and included only male students, as the MTC enrolls men exclusively; therefore, the findings may not generalize to female students or other military and educational settings. Future studies should use longitudinal designs and include larger, more diverse, multi-institutional samples. Fourth, social desirability bias within a military culture may have led to underreporting of stress symptoms. Moreover, the PSS-10 in this study demonstrated only moderate internal consistency (Cronbach’s α = 0.69). While this value falls within the acceptable range, it is lower than typically reported in international samples. This reduced reliability may have introduced some measurement error, potentially attenuating associations between stress and other variables. Cultural and linguistic factors could also play a role, and future research should consider re-validation of the scale in Omani populations to ensure stronger reliability. Additionally, the internal consistency values for some BFI subscales, particularly extraversion (α = 0.40) and openness (α = 0.59), were low in this study, which may limit the reliability of these predictors. This issue may reflect cultural and linguistic differences in how certain personality traits are expressed and interpreted in Omani or broader Arab contexts, as personality instruments developed in Western settings can show variable performance across different populations. Methodological factors, including the self-report nature of the survey and potential differences in item interpretation, may also have contributed. Future studies in Oman should consider validating or culturally adapting personality instruments and complementing them with qualitative methods to better capture complex personality constructs. Although LASSO regression was applied with cross-validation to reduce the risk of overfitting, the observed differences in model performance between the training and test datasets suggest that some degree of overfitting may still be present. This limitation indicates that the model’s predictive performance might not generalize equally well to other populations beyond the study sample. To mitigate this, future studies should consider larger, more diverse samples and the use of external validation datasets to further test the robustness and generalizability of the model.

## 5. Conclusions

This study integrates personality traits, depressive symptoms, and psychological well-being into a single LASSO regression-based predictive model of perceived stress among first-year Omani military cadets. The model accounted for approximately 40% of the variance in perceived stress, with neuroticism and depressive symptoms showing the strongest positive associations, while psychological well-being showed a negative association with perceived stress. Given the cross-sectional design, these findings should be interpreted as concurrent predictive associations rather than causal or directional relationships.

In practical terms, the findings may help inform early, multidimensional screening and mental health support within military educational settings. Screening for psychological vulnerability at induction, combined with targeted stress-management interventions, resilience training, culturally sensitive counseling, well-being education, and peer-support initiatives, may support cadet mental health during training. However, the findings should be interpreted cautiously in light of the single-institution, male-only sample and the low internal consistency observed for some Big Five subscales. Future studies should externally validate this model in larger, more diverse, and multi-institutional military student samples, use culturally validated measures with stronger psychometric properties, and employ longitudinal designs to examine temporal relationships, implementation feasibility, and cost-effectiveness.

## Figures and Tables

**Table 1:** Characteristics of the participants.

Characteristics	*N* = 266
Age (mean, range)	19.04 (17–24)
Highschool score (mean, range)	83.55 (59–100)
Chronic physical illness (n, %)	4 (1.50%)
Family history of mental illness (n, %)	10 (3.65%)
Past or current mental illness diagnosis (n, %)	4 (1.50%)
Current financial difficulties (n, %)	83 (31.20%)
Past alcohol misuse (n, %)	2 (0.75%)
Past drug misuse (n, %)	5 (1.88%)
Marital status	
Single	264 (99.24%)
Divorced	1 (0.38%)
Widowed	1 (0.38%)

**Table 2:** Descriptive Statistics.

	Mean	Standard Deviation	Skewness	Kurtosis
PSS ^a^	15.18	5.80	0.10	0.59
WHO ^b^	15.71	5.48	–0.52	–0.05
PHQ ^c^	8.02	5.46	0.86	0.39
Extraversion	19.31	4.06	–0.18	–0.42
Agreeableness	33.61	5.77	–0.13	–0.57
Conscientiousness	31.29	5.75	0.18	–0.56
Neuroticism	20.18	5.38	–0.13	–0.35
Openness	26.57	5.78	–0.54	0.16

^a^ Perceived Stress. ^b^ Well-being. ^c^ Patient Health Questionnaire.

**Table 3:** Correlation in relation to Perceived Stress Scale (PSS) scores.

	Pearson Correlation	
	*r*	*p*
WHO ^a^	–0.53	<0.001
PHQ ^b^	0.46	<0.001
Extraversion	–0.29	<0.001
Agreeableness	–0.23	<0.001
Conscientiousness	–0.38	<0.001
Neuroticism	0.56	<0.001
Openness	–0.08	0.18

^a^ Well-being. ^b^ Patient Health Questionnaire.

**Table 4:** Stability of LASSO regression coefficients across 100 runs.

	Mean	Standard Deviation	Selection Frequency
WHO ^a^	–0.32	0.04	100%
PHQ ^b^	0.15	0.04	100%
Extraversion	–0.03	0.04	39%
Agreeableness	0.04	0.05	44%
Conscientiousness	–0.02	0.03	42%
Neuroticism	0.35	0.05	100%
Openness	0.01	0.02	32%

^a^ Well-being. ^b^ Patient Health Questionnaire.

**Table 5:** Predictive performance of the full LASSO model and reduced sensitivity model.

Model	Test R^2^	MSE	RMSE	MAE
Full LASSO model	0.40 ± 0.08	20.24 ± 2.48	4.49 ± 0.28	3.61 ± 0.23
Sensitivity model (PHQ, WHO, and neuroticism)	0.41 ± 0.08	19.85 ± 2.50	4.45 ± 0.28	3.57 ± 0.23

## Data Availability

The data supporting the findings of this study are available from the corresponding authors upon reasonable request and with the permission of the local ethics committee.
